# Elevational Melanism in East African Honey Bees Involves a Major-Effect Locus

**DOI:** 10.1093/gbe/evag260

**Published:** 2026-10-14

**Authors:** Marco Mazzoni, Florian Loidolt, Sonja Kersten, Mark Otieno, Henry Njovu, Julius Vincent Lasway, Ricarda Scheiner, Martin Hasselmann

**Affiliations:** Department of Livestock Population Genomics, Institute of Animal Science, University of Hohenheim, Stuttgart 70599, Germany; Behavioral Physiology and Sociobiology, Biocenter, Julius-Maximilian-Universität Würzburg, Würzburg 97974, Germany; Department of Livestock Population Genomics, Institute of Animal Science, University of Hohenheim, Stuttgart 70599, Germany; Department of Water and Agricultural Resource Management, University of Embu, Embu 60100, Kenya; National Center for Wildlife, Protected Areas Directorate, Riyadh 12411, Kingdom of Saudi Arabia; Department of Wildlife Management, College of African Wildlife Management, Moshi 3031, Tanzania; Behavioral Physiology and Sociobiology, Biocenter, Julius-Maximilian-Universität Würzburg, Würzburg 97974, Germany; Department of Livestock Population Genomics, Institute of Animal Science, University of Hohenheim, Stuttgart 70599, Germany; KomBioTa—Center for Biodiversity and Integrative Taxonomy, University of Hohenheim and State Museum of Natural History, Stuttgart, Germany

**Keywords:** adaptation, chromosomal inversion, CRISPR/Cas9, melanism

## Abstract

Thermal melanism, whereby darker pigmentation occurs in colder environments, is a widespread pattern; yet, its genetic and physiological basis in eusocial insects remains poorly understood. East African honey bees (*Apis mellifera*) occupy elevational gradients in which highland populations are darker, larger, and more cold-tolerant than lowland conspecifics. Here, we integrated population genomics, structural variations, and functional genomics to investigate elevational melanism. We sampled, phenotyped, and resequenced 139 workers from five East African mountain systems. A genome-wide association study identified 977 single-nucleotide polymorphisms (SNPs) significantly associated with abdominal pigmentation, with a single peak at the *ebony* ortholog *AmEbony* on chromosome 1. Allelic variation at *AmEbony* formed three genetic clusters tracking a continuous pigmentation gradient, and 77 divergent SNPs (*D*_XY_ = 1) almost perfectly discriminated dark from light bees. Two inversions (r7, r9), previously linked to high-elevation adaptation, were detected across additional mountain systems and enriched in dark, highland genomic backgrounds but did not replace *AmEbony* as the primary pigmentation locus. CRISPR/Cas9 disruption of *AmEbony* in *A. mellifera carnica* produced darker adults in a qualitative comparison, supporting a conserved role in pigmentation. Head RNA-seq and GENIE3 analysis identified recurrent genotype-associated modules involving oxidoreductase activity and odorant binding. Because population structure covaries with elevation and pigmentation, the GWAS supports *AmEbony* as a major-effect locus but cannot identify causal variants. Similarly, head-expression data identify molecular changes associated with *AmEbony* disruption but cannot establish whether they contribute to pigmentation or whether naturally segregating East African alleles act through the same pathways.

Significance This study resolves a central mystery in evolutionary biology: how complex organisms architect adaptation to extreme environments. By integrating population genomics, quantitative pigmentation phenotyping, and CRISPR/Cas9 functional perturbation in the East African honey bee, we demonstrate that elevational melanism is driven by a major-effect architecture centered on the *AmEbony* locus. Disruption of *AmEbony* supports a role in honey bee pigmentation and is associated with head transcriptional changes involving oxidoreductase activity and odorant binding. These findings suggest hypotheses about additional functions but do not establish effects on chemosensory performance, detoxification, redox physiology, or survival. Our findings move beyond correlation to provide an experimentally validated framework for understanding how structural genomic variation and master regulatory genes coordinate the systemic physiological reprogramming required for high-altitude colonization in a globally vital pollinator.

## Introduction

Adaptation to heterogeneous environments is a central driver of evolutionary divergence and speciation. In ectotherms, two key traits, body pigmentation and body size, are critical for coping with the thermal and physiological challenges imposed by elevational gradients. The thermal melanism hypothesis (TMH) proposes that darker pigmentation enhances solar-heat absorption and thus confers a selective advantage in cooler, high-altitude environments (Clusella Trullas et al. 2007; Clusella-Trullas et al. 2008; Clusella-Trullas and Nielsen 2020). In parallel, larger body size can increase thermal inertia and improve performance under low-temperature conditions (Stevenson 1985). Empirical support for TMH has accumulated across a broad range of insect orders. Among Lepidoptera, Pieridae and Nymphalidae species exhibit increasingly dark wing patterns with increasing altitude (Kingsolver 1985; Markl et al. 2022), a pattern corroborated by large-scale surveys of North American butterflies across Lycaenidae, Pieridae, Nymphalidae, and Papilionidae (Stelbrink et al. 2019). Comparable elevational and seasonal clines in pigmentation have been reported in Odonata (Novella-Fernandez et al. 2023), Orthoptera (Harris et al. 2013), Diptera (Freoa et al. 2023), and Hymenoptera, including paper wasps (de Souza et al. 2020). Analogous patterns are evident in vertebrates: color polymorphism in the rock pocket mouse (Nachman et al. 2003; Hoekstra et al. 2004) and Peromyscus mice (Barrett et al. 2019; Hager et al. 2022) are tightly associated with environmental heterogeneity and local adaptation. Together, these studies highlight thermal melanism as a widespread and powerful mechanism facilitating adaptation to high-altitude and other thermally challenging environments. However, a pigmentation–elevation correlation does not demonstrate thermal selection. In sub-Saharan *Drosophila melanogaster*, pigmentation increased with altitude, but ultraviolet radiation was the strongest ecological predictor, suggesting that UV protection, rather than temperature alone, may also contribute to elevational melanism (Bastide et al. 2014)

The western honey bee, *Apis mellifera*, is well suited for studying genomic differentiation across climatic gradients (Wallberg et al. 2014). In East African mountain systems, montane bees historically assigned to *Apis mellifera monticola* occur in cool, humid habitats, whereas *Apis mellifera scutellata* is associated mainly with warmer lowlands and savannahs (Ruttner 1988). Because these populations share most of their genomes and can be admixed, we use the operational terms highland and lowland throughout and reserve subspecies names for their taxonomic context. Highland bees exhibit several distinguishing characteristics. They are darker in color, larger in size, and capable of flight at lower temperatures (Ruttner 1988; Gruber et al. 2013; Wallberg et al. 2017), consistent with the TMH. Population-genomic analyses of Kenyan honey bees showed that *A. m. monticola* and *A. m. scutellata* share approximately 98.5% of their genomes, with pronounced differentiation concentrated in chromosomal inversions r7 and r9 (Wallberg et al. 2017; Christmas et al. 2019). Recent work has confirmed these inversions in Ugandan honey bee populations and linked them to elevation-related gene expression differences, particularly in stress tolerance and cuticular hydrocarbon pathways (Mazzoni et al. 2025). However, these inversions do not fully explain the observed variation in pigmentation. The present study aims to create a large genomic dataset derived from honey bees from East African mountain systems and test abdominal pigmentation directly across the combined panel.

Establishing causal links between specific genes and adaptive phenotypes requires functional validation (Barrett and Hoekstra 2011; Stern 2013). CRISPR/Cas9 genome editing has become a powerful approach for dissecting gene function in diverse organisms (Jinek et al. 2012). In *Drosophila*, *tan* and *ebony* knockouts (KOs) alter both melanin deposition and cuticular hydrocarbon profiles (Massey et al. 2019). *Ebony* is also expressed in nervous tissue and participates in dopamine and histamine metabolism; consequently, a complete KO can have broader effects than a tissue-specific natural pigmentation allele (Hovemann et al. 1998), including alterations of behavior (Pantalia et al. 2023). Natural pigmentation differences in *Drosophila ebony* can involve tissue-specific *cis*-regulatory elements (Pool and Aquadro 2007). In honey bees, targeted disruption of *yellow* (y) affects expression of key melanogenesis enzymes such as *laccase 2* and *Aralkylamine N-acetyltransferase*, with consequences for pigmentation and biogenic-amine metabolism (Arakane et al. 2005; Nie et al. 2021). CRISPR/Cas9 disruption can therefore test the conserved function of *AmEbony*, but it cannot by itself identify which linked natural variant is causal or reproduce the mechanism of a tissue-specific natural allele.

Here, we integrate population-genomic analyses, transcriptome analyses, and functional genomics to elucidate the genetic architecture and regulatory basis of high-altitude pigmentation in East African honey bees. We tested whether abdominal pigmentation maps to a major locus across several East African mountain systems and whether the altitude-associated r7 and r9 inversions are independently associated with pigmentation or mainly mark highland genomic background. We assembled and phenotyped population samples from multiple African mountain systems, the Rwenzori Mountains (Uganda), Mt. Kilimanjaro and Mt. Meru (Tanzania), and Mt. Kenya and Mt. Elgon (Kenya), and combined these data with genomic and color phenotypes from Wallberg et al. (2017). A genome-wide association study (GWAS) reveals a strong involvement of the gene *ebony* regarding abdominal pigmentation, with little effect determined from additional loci beyond the major inversion regions. Furthermore, to establish causality at a key candidate locus, we used CRISPR/Cas9 to generate *ebony* KOs in *Apis mellifera carnica* and performed tissue-specific RNA-seq on head, thorax, and abdomen. This dual approach, linking landscape-level allele-frequency patterns and expression shifts with precise functional perturbation, demonstrates how a gene perturbation reshapes gene regulatory networks and confirms a causal role of *AmEbony* in honey bee pigmentation.

Collectively, our study identifies a major pigmentation-associated locus, tests the conserved pigmentation function of *AmEbony*, and characterizes recurrent head-expression consequences of gene disruption. The study distinguishes population association, gene-level functional evidence, and downstream KO effects in a very important ecological species, while acknowledging the limitations imposed by linkage, population structure, pleiotropy, and tissue specificity.

## Materials and Methods

### WGS and Analysis

DNA was extracted from 76 full thorax from one honey bee per colony using the CTAB protocol (Rusterholz et al. 2015) and processed to 2 × 150-bp paired-end sequencing (Illumina Novaseq X) targeting 25× coverage (Biomarker Technologies (BMK) GmbH). To get a more comprehensive GWAS concerning the pigmentation of honey bees, we integrated the newly sequenced samples into a larger dataset, including additional East African honey bee publicly available whole-genome data. We included 24 highland and lowland honey bees collected in Uganda, sequenced with a mean coverage of 25× (Mazzoni et al. 2025) (Bioproject: PRJNA1179698), and 39 highland and lowland honey bees sampled in Kenya in two different regions (Wallberg et al. 2017) (Bioproject: PRJNA357367), all with 12× mean coverage. SNP calling was carried out with the very same pipeline as in (Mazzoni et al. 2025). We used “HaplotypeCaller” from gatk4 v4.3.0.0, followed by joint genotyping using the “GenotypeGVCFs” function (der Auwera et al. 2013; Poplin et al. 2017). We imported data in a batch size of 20 by chromosome with “GenomicsDBImport.” The resulting VCFs were merged with bcftools v1.11 (Danecek et al. 2021). Allele balance for each variant was annotated with gatk3 v3.8.1.0 and filtered using vcffilter with the condition “ABHet > 0.25 & ABHet < 0.75 | ABHet < 0.01,” to allow for homozygous variants, too (Garrison et al. 2022). After evaluating the distribution of the parameters from the INFO field in the final VCF, additional filters were applied with bcftools view v1.11 (Danecek et al. 2021). The filtering criteria for variant sites included the following thresholds: MQ < 40, MQRankSum < −5, MQRankSum > 5, ExcessHet > 20, AC = 1, INFO/DP < 11660, INFO/DP > 21654, AN < 826, FS > 20, SOR > 5, and QD < 5. Only biallelic SNPs were considered, and indels were excluded. The gatk4 function “SelectVariants” was used to retain only variant sites in chromosomes for downstream analysis (der Auwera et al. 2013). Finally, the remaining variants were phased using beagle v5.4 (22Jul22.46e.jar) (Browning et al. 2018, 2021).

### Honey Bees Phenotyping, GWAS, and Population-Genomic Statistics

A GWAS was performed to investigate the genetic basis of variation in abdominal pigmentation. The analysis used both the WGS data generated in this study and publicly available genomic data for the same species. Individual abdomens were imaged using a ZEISS Stemi 305 stereomicroscope equipped with an Axiocam 105 color camera (ZEISS, Germany). Pigmentation was scored from the resulting digital images for abdominal tergites 2, 3, and 4 using a scale adapted from Dembeck et al. (2015). Each tergite was assigned a score ranging from 0 (completely dark) to 1 (completely yellow), in increments of 0.125. The three tergite scores were summed to obtain the SoY phenotype across the scored abdominal segments, ranging from 0 to 3, with lower values indicating darker overall abdominal pigmentation. The SoY score was analyzed as a quantitative response phenotype using the univariate linear mixed model implemented in GEMMA (Zhou and Stephens 2012). For each test, SNP dosage was fitted as the fixed effect of interest, while a genomic relatedness matrix was included as a random effect to account for population structure and cryptic relatedness. No additional fixed-effect covariates were included beyond the model intercept. Each SNP was tested individually, and statistical significance was evaluated using the Wald test. The resulting *P*-values were adjusted for multiple testing using the BH FDR test, with an adjusted *P*-value of 0.05. To further characterize genomic variation in relation to pigmentation phenotypes, we computed standard population-genetic statistics: nucleotide diversity (*π*), fixation index (*F*_ST_), and absolute genetic divergence (*D*_XY_) using pixy (Korunes and Samuk 2021). Calculations were performed with the flags --stats pi fst dxy and a window size of 1 bp, thereby providing site-wise resolution.

### CRISPR/Cas9 Experiment Design

The phylogenetic tree to assess the *AmEbony* orthology to the *D. melanogaster* ebony gene was performed using iQtree2 running on 12 threads, performing ultrafast bootstrap with 1000 replicates and allowing the tool to find the best evolutionary model with the -m MFP flag (Minh et al. 2020). The honey bee pigmentation-related gene *ebony* (NCBI: LOC409109/GB46429) is located on chromosome 1 and comprises 15 exons. We compared the three NCBI transcript variants and found that they encode identical amino acid sequences. Therefore, we used the longest transcript (XM_026445876.1) for sgRNA target selection. Candidate crRNA targets were identified in Benchling (Benchling 2023) following Değirmenci et al. (2020): 20-bp protospacers with an NGG PAM and a 5′ guanine. We prioritized guides with high predicted cutting efficiency (on-target >50) and high specificity (low off-target risk). Candidate sites were additionally validated with SPLIGN (Kapustin et al. 2008) to ensure they lay entirely within an exon and with Vienna RNAfold (Lorenz et al. 2011) to confirm appropriate sgRNA secondary structure and accessibility for Cas9 interaction. To maximize functional disruption, we favored targets positioned early in the *ebony* open reading frame (within ∼800 bp of a 2583-bp ORF). The three best guides were synthesized in-house via overlapping Phusion PCR to generate the DNA template, followed by T7 in vitro transcription, sgRNA purification, and quantification as described in Değirmenci et al. (2020). Pilot injections across multiple sgRNA concentrations were used to estimate both larval hatching success and mutation frequency, which were combined into a “mutant rate” to rank treatments. The best-performing guide (5′-GCAGGAATTACCTTATGCAG-3′; ORF position 635 on the plus strand) was selected for the main experiment at 92 ng/µL sgRNA with 3.13 µM Cas9, delivering 400 pL per embryo. Embryos were collected from *A. m. carnica* colonies using Jenter systems and injected within 0 to 1.5 h after oviposition to target the single-cell stage. Artificial rearing of the honey bees in the laboratory followed established protocols (Schmehl et al. 2016; Değirmenci et al. 2020). Adult workers were phenotyped at 5 d postemergence, and mutations were confirmed by fluorescently labeled PCR spanning the target site and capillary electrophoresis, comparing amplicon length shifts to the 171-bp WT fragment and by qualitative visual comparison with WT controls; no quantitative pigmentation estimate was obtained.

### RNA Extraction, Library Preparation, and Sequencing

For the functional transcriptomic analysis of *AmEbony*, we collected 12 individual bees, comprising 6 Eby^+^/Eby^+^ (WT) and 6 Eby^−^/Eby^−^ (KO) individuals. From each bee, we dissected and processed three distinct tissues: head, thorax, and abdomen. To investigate potential tissue-specific transcriptional responses to the loss of *AmEbony* function, total RNA was extracted from each tissue sample using TRIzol reagent (Thermo Fisher Scientific) according to the manufacturer's guidelines with minor optimizations for honey bee tissues. In brief, each sample was homogenized in 500 μL of TRIzol, followed by phase separation with 100 μL of chloroform and precipitation with 300 μL of isopropanol. After centrifugation and solvent removal, the RNA pellet was resuspended in 30 μL of RNase-free water and stored at −80 °C until further processing. The integrity and concentration of the RNA were assessed prior to library preparation, followed by standard barcoding procedures for multiplexing and sequencing on an Illumina NovaSeq 6000 platform with a 2 × 150-bp paired-end configuration. Each library generated approximately 40 million high-quality reads per sample, ensuring sufficient coverage for quantitative transcriptome profiling across all three tissue types.

For transcriptome sequencing, the read quality was checked using fastqc (Andrews 2010). Adapters were removed, and reads were trimmed using Trimmomatic v0.36 (Bolger et al. 2014). After trimming, fastqc was used again to check the quality of the trimming process. Trimmed reads were used by Kallisto (Bray et al. 2016) to quantify the abundances of transcripts. Then, DESeq2 (Love et al. 2014) was used to analyze DEGs.

### Gene Regulatory Network Construction and Analysis (GENIE3)

To investigate the transcriptional architecture associated with AmEbony function, we constructed a gene regulatory network using the GENIE3 algorithm (Huynh-Thu et al. 2010). The network inference was performed on the variance-stabilized expression matrix derived from the RNA-seq data of Eby^+^/Eby^+^ (WT) and Eby^−^/Eby^−^ (KO) individuals. Prior to network construction, genes with consistently low expression were removed, and the remaining genes were variance-stabilized to reduce heteroscedasticity across samples.

## Results

### Identifying Genomic Regions Linked to Pigmentation Differentiation

We analyzed 139 honey bees sampled across five major East Africa mountain systems that span pronounced elevational gradients: Mount Elgon in western Kenya (maximum elevation ∼4,321 m; maximum sample elevation 2600 m), the Rwenzori Mountain system in Uganda (maximum elevation ∼5,109 m: maximum sample elevation: ∼2700 m), Mount Kenya in central Kenya (maximum elevation ∼5,199 m; maximum sample elevation: ∼2800 m), Mount Kilimanjaro in northern Tanzania (maximum elevation ∼5,895 m; maximum sample elevation: ∼2800 m), and Mount Meru in northern Tanzania (maximum elevation ∼4,566 m; maximum sample elevation ∼1650 m; Table S1). These systems were selected because they encompass steep climatic gradients over short geographic distances and harbor natural populations of *A. mellifera* adapted to contrasting thermal and ecological conditions.

Each individual was assigned a quantitative abdominal pigmentation score (Fig. S1), derived from standardized, high-resolution digital images obtained under controlled laboratory conditions, thereby minimizing phenotyping bias. Abdominal pigmentation was associated with elevation (Fig. 1a), with high-altitude bees exhibiting markedly darker abdomens than their lowland counterparts. However, since bees were sampled from different locations, this may reflect genetic differences, phenotypic plasticity, or both.

**Fig. 1.**
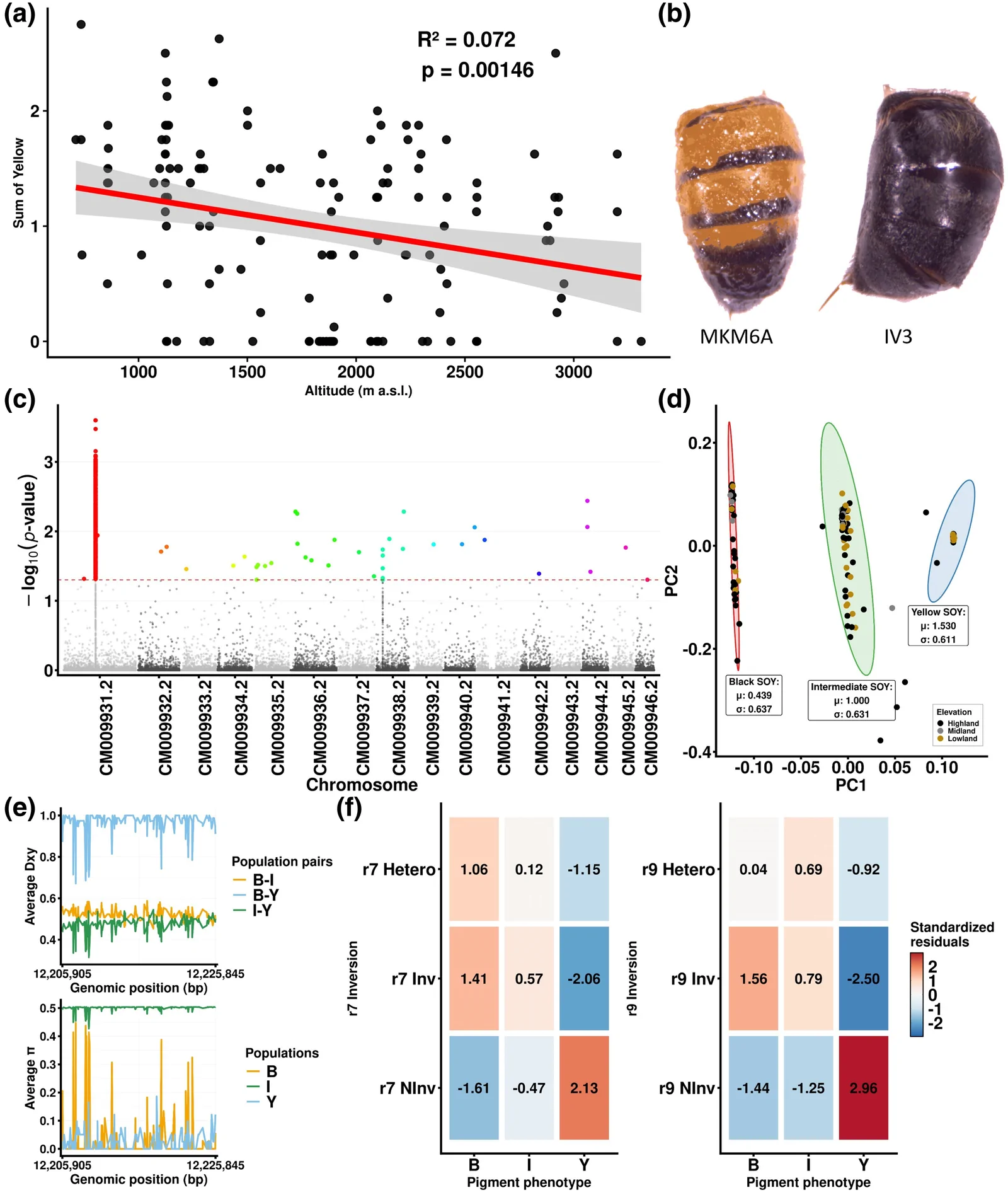
Altitudinal variation in pigmentation and associated genomic signals. a) Relationship between altitude (m a.s.l.) and the pigmentation index SOY: the yellow component decreases with increasing altitude (red regression line; gray band = confidence interval). *R*^2^ and *P*-value are reported. b) Representative individuals from the two extreme phenotype clusters (yellow vs. black abdomen). c) Genome-wide association (Manhattan) plot for the pigmentation phenotype: *y*-axis shows −log10(*P*); the dashed line indicates the significance threshold and highlighted points denote the strongest association signals. d) PCA-based clustering of individuals using SOY-related metrics, showing three groups (black—B, intermediate—I, yellow—Y ellipses) and their altitudinal origin; insets report group summary statistics (mean μ and standard deviation σ). e) Population-genetic statistics across the candidate interval: mean divergence (*D*_XY_) between phenotype–group pairs (B–I, B–Y, I–Y) and nucleotide diversity (*π*) within each group along genomic coordinates. f) Association between pigmentation phenotype (B/I/Y) and inversion genotypes (heterozygous, inversion homozygous, noninversion homozygous) for r7 and r9: heatmaps show standardized residuals (red = enrichment relative to expectation; blue = depletion).

Whole-genome sequencing (WGS) of the 139 individuals initially identified approximately 10 million single-nucleotide polymorphisms (SNPs). After stringent quality filtering based on read coverage, minor allele frequency, and missing data (see Material and Methods), we retained 6,631,751 high-quality SNPs for downstream analyses. Because abdominal pigmentation could not be scored for one individual, the final association analyses were conducted on 138 phenotyped bees (Table S2). This curated SNP set provided the basis for all subsequent genome-wide association and population-genomic analyses.

We performed a GWAS using the univariate linear mixed model implemented in GEMMA, individually testing 6,631,751 SNPs. The continuous Sum of Yellow (SoY) score was used as the response phenotype, SNP dosage was fitted as the fixed effect of interest, and a genomic relatedness matrix was included as a random effect to account for pairwise relatedness and population structure. No additional fixed-effect covariates were included beyond the intercept. In the null model, GEMMA estimated a PVE of 0.9999 ± 0.0067, with a genetic variance of vg = 1.8885, a residual variance of ve ≈ 1.9 × 10^−5^, a log restricted maximum likelihood of −90.89, and a log maximum likelihood of −86.36. These values indicate that the fitted model assigned almost all phenotypic variance to the genomic random effect. However, this boundary estimate should not be interpreted as evidence that abdominal pigmentation is fully heritable, because the genomic random effect can also capture phenotypic covariance associated with relatedness and population structure. Environmental and developmental contributions to pigmentation therefore cannot be excluded.

Association statistics were corrected for multiple testing using the Benjamini–Hochberg (BH) false discovery rate (FDR) procedure (adjusted *P* < 0.05). We identified 977 SNPs significantly associated with abdominal pigmentation, the overwhelming majority of which were concentrated within a discrete region on chromosome 1 (ID: CM009931.2). The Manhattan plot (Fig. 1b) revealed a single, pronounced association peak spanning positions 12,205,905 to 12,225,845 bp, with a dense cluster of significant variants. Although the dense cluster of significant markers and elevated *D*_XY_ supports the presence of a pigmentation-associated haplotype, the strong linkage within the interval precludes attributing the association to specific variants. A limited number of additional loci located on other chromosomes exhibited moderate association signals but did not surpass the genome-wide significance threshold.

The zoomed-in view of the Manhattan plot (Fig. S2) further resolves the lead SNPs within this region, revealing a region dense with strongly associated variants. Together, these patterns indicate that the altitudinally associated variation in abdominal pigmentation among East African honey bees is dominated by a single genomic locus, with relatively minor contributions from the remainder of the genome. This contrasts with the largely polygenic architecture expected for many quantitative traits and highlights a major-effect locus driving elevational melanism in this system.

To assess the robustness of the association signal to the construction of the genomic relationship matrix, we additionally implemented a leave-one-chromosome-out (LOCO) linear mixed model. In this framework, when testing SNPs on chromosome i, the genomic relationship matrix is re-estimated excluding all variants on chromosome i, so that local effects are not absorbed into the random effect term. Re-running the GWAS under the LOCO scheme with the same set of individuals, genotypes, and covariates yielded a concordant major peak on chromosome 1, confirming the robustness of the signal (Figs. S3 and S4). However, the LOCO approach does not eliminate potential confounding caused by genome-wide ancestry that covaries with both elevation and pigmentation.

Within this associated region, several annotated gene models are present. Reciprocal BLASTP searches between *Drosophila melanogaster ebony* (FBgn0000527) and the *A. mellifera* candidate LOC409109 yielded concordant top hits in both directions (*E* = 0.0; bit score = 798; 48% identity over 883 aligned amino acids; 67% positives; 3% gaps), strongly supporting LOC409109 as the *A. mellifera* ortholog of *ebony*. To further validate orthology and rule out paralogy, we reconstructed a phylogeny of *ebony* homologs across hymenoptera order. The *A. mellifera* sequence clustered with Apoidea e*bony* homologs and showed, as expected, a high evolutionary divergence to *D. melanogaster* (Fig. S5, Table S3). For clarity of presentation, we refer to this locus as “*AmEbony*” throughout the manuscript.

To characterize the genetic structure at *AmEbony*, we extracted all GWAS-significant SNPs located within this locus and summarized their allelic variation using a principal component analysis (PCA). This analysis revealed three genetically distinct clusters (Fig. 1d) that mapped closely onto the observed abdominal pigmentation phenotypes. One cluster comprised predominantly darkly pigmented (black) bees with an average pigmentation score of 0.439 ± 0.637, a second cluster comprised individuals with intermediate pigmentation with an average score of 1.00 ± 0.631, and a third cluster included predominantly lightly pigmented (yellow) bees with an average score of 1.53 ± 0.611. The close correspondence between the PCA-derived clusters and phenotypic pigmentation scores further supports a strong relationship between *AmEbony* haplotype variation and abdominal melanism. Finally, to assess whether the clustering at *AmEbony* reflects broader population structure, we compared the local PCA with a genome-wide PCA (Fig. S6). The genome-wide PCA showed partial clustering by sampling population, but this pattern differed from the three genetic clusters identified in the *AmEbony*-based PCA (Fig. 1d, Fig. S6). Thus, the local clustering differs from the main axes of genome-wide genetic variation.

To investigate the potential functional relevance of the associated variants, we annotated all significant SNPs within *AmEbony* using SnpEff (Cingolani et al. 2012). Although most significant SNPs were intronic, a subset was predicted to affect regulatory or coding regions. Specifically, we identified 3 SNPs located in the 3′ untranslated region (UTR), 13 SNPs in the 5′UTR, 13 missense substitutions, and 26 synonymous substitutions within the coding sequence. To evaluate variants outside the annotated *AmEbony* transcript, we extended the analysis to 10 kb on either side of LOC409109 and annotated the statistically significant SNPs using SnpEff (Cingolani et al. 2012). This identified 118 upstream and 11 downstream variants, showing that the associated interval extends into flanking noncoding sequence and includes potential *cis*-regulatory candidates. Together with the transcript-associated missense, UTR, and intronic variants, these annotations define several plausible functional classes through which variation at *AmEbony* may influence pigmentation. Because these variants occur within the same strongly linked interval, the present data cannot determine the relative contribution of regulatory and coding changes.

To identify the variants most strongly associated with pigmentation divergence among the three phenotypic groups, we calculated standard population-genomic statistics using pixy (Korunes and Samuk 2021), including *F*_ST_, *D*_XY_, and nucleotide diversity (*π*), across the pigmentation-defined clusters (yellow, intermediate, and black) (Fig. 1e). Group membership was determined using the local *AmEbony* PCA, with the three clusters labeled black, intermediate, and yellow according to their mean pigmentation scores. Similar genomic patterns were observed across the analyzed interval and group comparison for *F*_ST_ and *D*_XY_ (Table S4), with peaks occurring at corresponding genomic positions. Thus, regions showing high *F*_ST_ also showed elevated *D*_XY_. For downstream analyses, we focused on *D*_XY_, as it measures absolute genetic divergence between populations and is less sensitive to differences in within-population diversity. Using this approach, we identified 77 SNPs within *AmEbony* with *D*_XY_ = 1 between the predominantly black cluster and the other pigmentation groups. Among these highly divergent variants, two SNPs were located within the 5′UTR, five were synonymous, five were missense substitutions, and the remaining SNPs resided in intronic regions (Table S5). These results describe differentiation within the pigmentation-associated interval. Because the clusters and focal SNP set were defined using the association signal, this analysis cannot date their divergence or establish when an association with elevation arose.

Strikingly, these 77 SNPs almost completely discriminate black-pigmented bees from yellow-pigmented bees (Fig. 1d). Their allele frequencies shift gradually across the pigmentation spectrum: the alleles associated with dark pigmentation are rare in the yellow cluster, intermediate in frequency in the phenotypically intermediate cluster, and approach fixation in the black cluster. This allele-frequency cline mirrors the continuous phenotypic gradient in abdominal pigmentation and highlights strong, phenotype-correlated differentiation at *AmEbony*.

The allele frequencies reveal a close correspondence between distinct *AmEbony* haplotypes and the pigmentation gradient, strengthening the association between variation across this locus and abdominal pigmentation.

### Altitude-Associated Inversions r7 and r9 Covary With Pigmentation

Previous genomic studies of East African honey bees identified two large chromosomal inversions—hereafter r7 and r9—on chromosome 7 and chromosome 9, respectively, that are associated with local adaptation to high-elevation environments (Wallberg et al. 2014, 2017; Christmas et al. 2019; Mazzoni et al. 2025). In addition, a region of elevated genetic differentiation between highland and lowland populations has been reported at the proximal end of chromosome 5. Although this region shows patterns suggestive of an inversion, its structural status remains unresolved in the absence of long-read data; we therefore refer to it as the r5 region (Mazzoni et al. 2025). Our sampling included bees from Mount Kilimanjaro and Mount Meru in northern Tanzania, two high-elevation regions where the presence of r7 and r9 had been hypothesized based on ecological and altitudinal patterns but not directly demonstrated (Wallberg et al. 2017). We can now see that the allele frequency of the inverted allele increases with respect to the elevation in all three populations: Tanzanian, Ugandan, and Kenyan (Fig. S7). Genotyping of these Tanzanian samples confirmed the presence of both r7 and r9, providing the first formal documentation of these inversions in Kilimanjaro and Meru populations. This extends the known geographic range of these structural variants beyond previously characterized East African mountain systems (Figs. S8 and S9).

To examine the relationship between inversion genotype and local genetic structure, we assigned each of the 138 phenotyped bees to one of the three *AmEbony*-based genetic clusters, labeled black, intermediate, and yellow according to their predominant pigmentation. Independently, we assigned r7 and r9 inversion genotypes (homozygous for the reference orientation, heterozygous, or homozygous for the inverted orientation) using diagnostic SNPs (Wallberg et al. 2017; Christmas et al. 2019; Mazzoni et al. 2025). For the r5 region, individuals were characterized as carrying highland-like or lowland-like haplotypes based on previously reported diagnostic SNPs. We then compared the distribution of inversion genotypes across the pigmentation-defined clusters using contingency tables (Fig. 1f, Table S6). This analysis tests independence between the two classifications, rather than an independent effect of inversion genotype on the *AmEbony-*based genetic clusters. Chi-square tests rejected independence between the aforementioned clusters and inversion genotype for r7 (*χ*^2^ = 16.33, *P* = 0.0026) and r9 (*χ*^2^ = 23.02, *P* = 0.0001) (Fig. 1f, full contingency tables in Table S6). In contrast, the r5 region showed no such pattern: haplotype frequencies did not differ significantly among the pigmentation clusters (*χ*^2^ = 6.47, *P* = 0.167), despite pronounced highland–lowland differentiation at this locus (Mazzoni et al. 2025).

Taken together, these results show that the two previously characterized inversions, r7 and r9, which are demonstrated to be associated with high-elevation adaptation, are also enriched in the pigmentation cluster characterized by darker abdominal coloration. This pattern is consistent with a shared geographic and ecological context: high, cool environments favoring both inverted haplotypes and dark pigmentation rather than a direct causal effect of the inversions on the pigmentation phenotype itself. In contrast, the r5 region, despite its strong altitudinal differentiation, shows no analogous concordance with pigmentation clusters. These findings identify *AmEbony* as the principal pigmentation-associated locus in this panel, whereas r7 and r9 more likely mark the broader highland genomic background than exert direct effects on pigmentation.

### CRISPR/Cas9 Disruption Supports a Conserved Pigmentation Function of *AmEbony*

To test the role of *AmEbony* in abdominal pigmentation, we employed CRISPR/Cas9-mediated gene disruption. Although we are aware that this approach does not distinguish the effects of naturally segregating coding and regulatory variants, it allowed us to examine the pigmentation phenotype and transcriptional changes associated with *AmEbony* disruption. Guide RNAs were designed to target conserved exonic regions of *AmEbony*, with the aim of generating frameshift mutations predicted to abolish gene function. Ribonucleoprotein complexes of Cas9 and specific sgRNA were microinjected into one-cell stage honey bee embryos to induce targeted mutagenesis. Because genome editing is not yet feasible in the East African populations analyzed in our GWAS due to a lack of infrastructure, we conducted the experimental work in *A. m. carnica*, a widely used experimental subspecies that provides a tractable model for functional genetics. This design assumes conservation of the core melanization pathway across *A. mellifera* subspecies while acknowledging that regulatory context and standing genetic variation may differ.

Edited individuals were genotyped using fluorescence-labeled amplicon (FLA) analysis, enabling unambiguous discrimination between wild-type (WT) and KO genotypes (Fig. 2a). The CRISPR-mediated disruption of *AmEbony* produced indel mutations at the targeted exon sites, consistent with loss-of-function mutations in the encoded protein (Fig. 2b). Injection at the one-cell stage reduces, but does not eliminate, the possibility of somatic mosaicism; tissue-level mosaicism and potential off-target edits were not quantified. We successfully established both WT and *AmEbony* KO individuals within laboratory colonies (Fig. S10), providing a controlled framework to assess the direct phenotypic consequences of *AmEbony* disruption on abdominal pigmentation and to characterize associated changes in gene expression. CRISPR/Cas9-mediated disruption of *AmEbony* produced visibly darker adults than WT controls in a qualitative comparison (Fig. 2a/b, Fig. S10). To characterize the accompanying molecular changes, we subsequently performed tissue-specific RNA-seq followed by network analyses and identified coordinated expression changes (Fig. 2c) as described later.

**Fig. 2.**
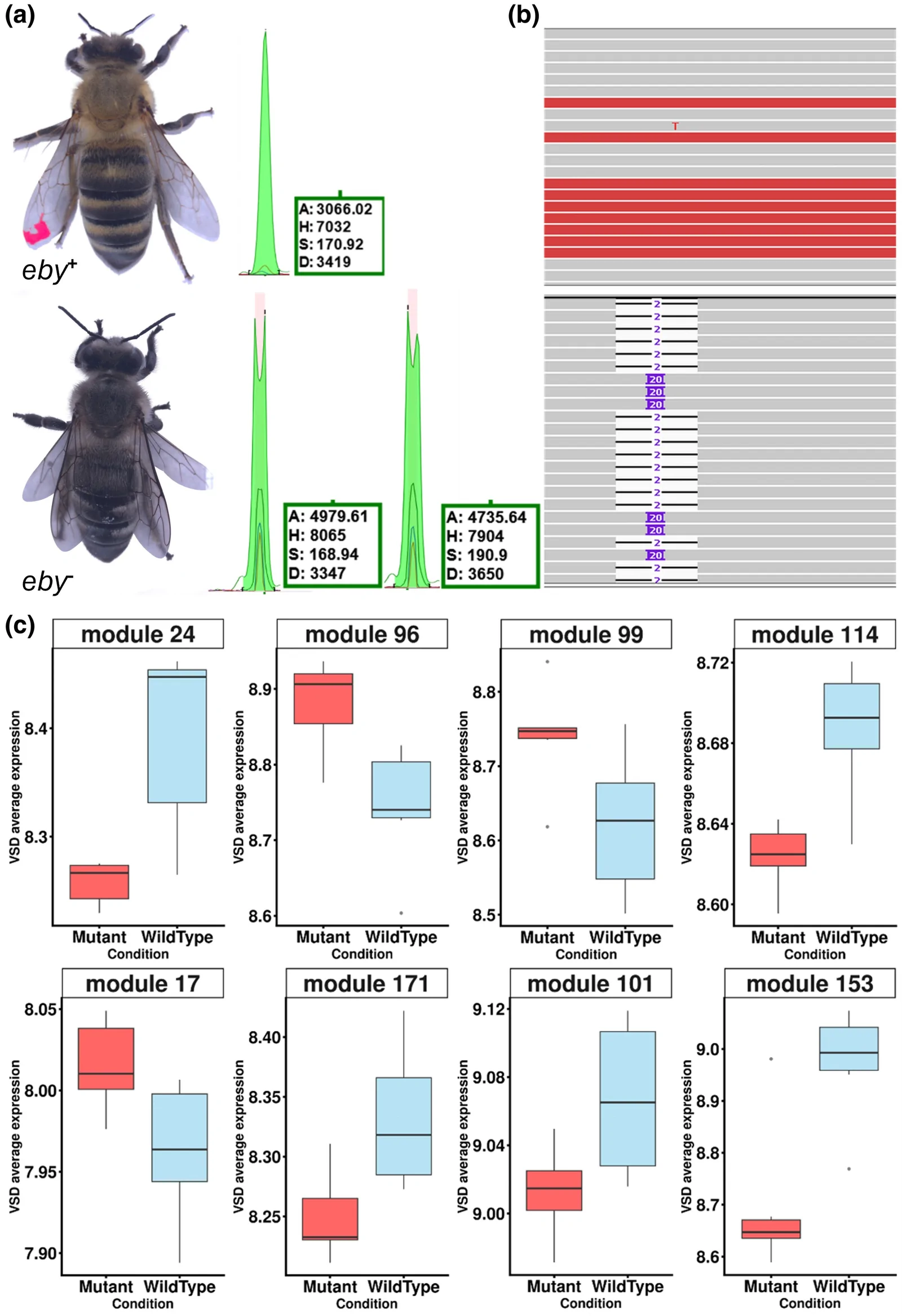
CRISPR/Cas9 validation of *ebony* editing and GENIE3-based transcriptomic module differences. a) A qualitative comparison of representative phenotypes of a wild-type individual (eby^+^, yellow pigmentation) and a CRISPR-edited knockout (eby^−^, dark pigmentation). Fragment length analysis (FLA) of the targeted locus (green electropherogram peaks) supports successful genome editing, with edited individuals showing fragment patterns consistent with indel formation at the CRISPR cut site. b) Read alignments across the targeted region (IGV-style view) further confirm editing at the expected locus, with recurrent indel classes (e.g. 2- and 20-bp events, indicated in purple). c) Gene regulatory network inference using GENIE3 (run 1) identified eight expression modules significantly associated with genotype (significance assessed within the GENIE3-based analysis workflow; *P*-values reported in Materials and Methods). Boxplots show mean (± standard deviation) variance-stabilized expression (VSD average expression) for each significant module (24, 96, 99, 114, 17, 171, 101, and 153) in mutant (red) versus wild-type (blue) samples.

### Tissue-Resolved RNA-seq and Head-Focused Coexpression Analysis

To explore how *AmEbony* loss of function affects transcriptional networks, we first performed differential expression analysis of head, thorax, and abdomen transcriptomes from WT and *AmEbony* KO bees. The pigmentation phenotype alone does not reveal the broader molecular consequences of AmEbony disruption. We therefore used RNA-seq to investigate associated changes in gene expression and identify potentially affected biological processes across tissues. Differentially expressed genes (DEGs) were detected only in the head transcriptome, whereas no DEGs were identified in the thorax and abdomen. We therefore focused the network analysis on the head-expression data, which identified ∼175 modules, providing a complementary view of coordinated expression patterns associated with *AmEbony* disruption.

To investigate coordinated expression patterns beyond individual DEGs, we used GENIE3 to infer a network from the head-expression data and identify gene modules and biological functions associated with *AmEbony* disruption. GENIE3 relies on tree-based ensemble regressors (Random Forests/Extra-Trees). Therefore, some stochasticity in edge inference was expected. To evaluate the robustness of the inferred functional signals, we repeated the full GENIE3 analysis five times using different random seeds and increased the number of trees from 1,000 to 2,000 per run. We then performed Gene Ontology (GO) enrichment analyses for each run and summarized enrichment reproducibility at the level of GO-term presence/absence per run. Across runs (assessing enrichment reproducibility at the level of “presence in a run,” i.e. detection in at least one module within that run), we observed 102 unique enriched GO terms. Only oxidoreductase activity (GO:0016491) was recovered in all five runs, whereas odorant binding (GO:0005549) was recovered in four of five runs (Table S7). Most other terms appeared in only one run. We therefore focused subsequent interpretation on these two recurrent functional categories, which represented the most stable enrichment signals across the seed-specific network reconstructions. Because all runs were generated from the same biological dataset, recurrence was considered a measure of computational stability rather than independent biological replication.

WT-biased modules were enriched for mitochondrial and energy-metabolism functions, including oxidative phosphorylation, respiratory electron transport, ATP metabolic processes, and mitochondrial complex assembly. KO-biased modules were instead enriched for redox-related functions, with oxidoreductase activity (GO:0016491) being the only GO term recovered in all five network runs (Table S7). Odorant-binding protein genes were also represented among the head DEGs, and odorant binding (GO:0005549) was recovered at the network-module level in four out of five runs. Although the DEG set as a whole showed no significant GO enrichment, the recurrence of odorant binding across network reconstructions identifies it as a consistent component of the head transcriptional response.

Together, these results provide a coherent molecular profile of *AmEbony* loss of function in head tissue, involving mitochondrial energy metabolism, redox-related activity, and odorant-binding genes. They extend the functional characterization of the KO beyond its visible pigmentation phenotype, while the relationship between these transcriptional changes and the naturally segregating East African *AmEbony* haplotypes remains to be tested.

## Discussion

East African honey bees occupy steep elevational gradients in which temperature, humidity, and solar irradiance change predictably over short spatial scales. Such systems provide a powerful natural laboratory for testing how pigmentation variation aligns with thermal ecology and how genome structure constrains or facilitates adaptation. Integration of population sampling across multiple mountain systems was employed to conduct a GWAS of abdominal pigmentation. This approach also involved structural-variant genotyping of two large altitude-associated haplotype blocks (r7, r9) and CRISPR/Cas9-induced disruption of *AmEbony* gene function, followed by transcriptome- and network-level analyses. These analyses address distinct levels of evidence: population association identifies an *AmEbony*-containing haplotype, CRISPR/Cas9 tests conserved gene function, and head RNA-seq characterizes downstream effects of gene disruption. The integrated design enabled us to investigate two related questions: first, whether a major-effect locus can exert disproportionately large phenotypic effects related to pigmentation variation and, second, whether *AmEbony* disruption produces broader transcriptional effects in head tissue, particularly across chemosensory, metabolic, and redox-related pathways.

### A Major Pigmentation Association-Centered on *AmEbony*

Our GWAS revealed a single pronounced association peak for abdominal pigmentation centered on *AmEbony*; evidence for additional minor-effect loci was limited. The concentration of the detectable association signal at this locus supports *AmEbony* as the main genomic contributor to pigmentation variation in this panel.

Population-genomic surveys in *A. mellifera* have documented extensive standing genetic variation and repeated signatures of local adaptation along environmental gradients (Gruber et al. 2013; Wallberg et al. 2017). Against this background, our finding that pigmentation variation is overwhelmingly concentrated at *AmEbony* supports a major-effect locus model, whereas the present data cannot establish contributions from a broader network of small-effect loci.

### *AmEbony* Dominates the Pigmentation Association Signal

The GWAS signal for abdominal pigmentation was strongly concentrated at *AmEbony*, with little evidence for additional loci of comparable effect. This concentration is biologically plausible because *ebony* catalyzes the conjugation of dopamine and β-alanine to produce *N*-β-alanyl dopamine, a central branch of insect cuticle pigmentation (Hovemann et al. 1998; Wittkopp et al. 2002; Massey and Wittkopp 2016). Variation affecting the expression or activity of such a biochemically central enzyme can produce substantial differences in cuticle coloration. Consistent with this expectation, the associated SNPs form a dense linked haplotype block spanning the *AmEbony* interval and its flanking sequence, and the three haplotype clusters closely track the observed pigmentation gradient. The broader *AmEbony* haplotype therefore represents the principal unit of association in this panel.

The frequency of the dark-associated *AmEbony* haplotype increased with elevation. Specifically, 66% of honey bees within this cluster were sampled from highland elevation, whereas this proportion decreased to 52% in the intermediate cluster and dropped to 37% in the yellow cluster. The direction of this cline is consistent with the TMH and with broader evidence linking darker coloration to thermal performance under cool environmental conditions (Clusella-Trullas et al. 2007; Clusella-Trullas and Nielsen 2020). Temperature-dependent fitness was not measured directly, and other elevation-associated factors, including ultraviolet radiation and desiccation, may also contribute to the pigmentation cline. Nevertheless, the combined association and haplotype results place *AmEbony* at the center of pigmentation differentiation along the elevational gradient and identify it as a major-effect locus for abdominal pigmentation in these populations.

### r7 and r9 Inversion Polymorphisms Are Associated With Pigmentation But Likely Act via Genomic Background

We confirm that the large haplotype blocks r7 and r9, previously inferred as inversions and tied to high-altitude adaptation (Wallberg et al. 2017; Mazzoni et al. 2025), are present not only in previously studied regions but also on Mount Kilimanjaro and Mount Meru. This extends the geographic range of these structural variants and strengthens the case that they are repeatedly recruited in high-elevation ecotypes. Inversion genotypes at r7 and r9 are nonrandomly associated with pigmentation clusters: inverted (highland) haplotypes are overrepresented among darker bees, whereas the r5 region shows no such pattern. This suggests that r7 and r9 do not directly encode pigmentation changes but rather maintain highland genomic backgrounds on which dark *AmEbony* alleles segregate. *AmEbony* remains the principal pigmentation-associated locus, whereas r7 and r9 define the genomic background of highland honey bees, thus requiring further testing.

### CRISPR/Cas9 Supports a Conserved Pigmentation Function of *AmEbony*

Our CRISPR/Cas9 experiments in *A. m. carnica* provide direct functional evidence that *AmEbony* supports a conserved role in honey bee pigmentation. Targeted disruption of *AmEbony* produced clear pigmentation changes, consistent with the locus acting as a key node in melanin biosynthesis and strongly supporting its identification as the major GWAS peak. Although rescue experiments are not possible in the honey bee yet, our results align with prior CRISPR-based manipulations of pigmentation and sensory genes in *A. mellifera* (e.g. *yellow* and *orco*). The feasibility of genome editing in *A. mellifera* moves the field beyond correlative association toward mechanistic dissection, enabling future tests of gene function, pleiotropy, and candidate variants (Hu et al. 2019; Chen et al. 2021; Nie et al. 2021).

### Gene Expression Consequences of *AmEbony* Disruption

Head transcriptomes from *AmEbony* KOs showed consistent shifts in modules enriched for odorant binding and oxidoreductase activity. These results are mechanistically coherent: in *Drosophila*, *ebony* is expressed in glia and cuticle, controls histamine/dopamine conjugation, and couples tanning, neuromodulation, and behavior; *ebony/tan* edits reshape cuticular hydrocarbons, which act as chemosensory cues (Hovemann et al. 1998; Hartwig et al. 2014; Izoré et al. 2019; Massey et al. 2019). In honey bees, an expanded odorant-receptor repertoire and specialized receptors for queen pheromones highlight the centrality of olfaction (Robertson and Wanner 2006; Wanner et al. 2007; Vernier et al. 2019). The observed redox and odorant-binding patterns may therefore reflect broad neural or metabolic consequences of disrupting dopamine/biogenic-amine metabolism. Oxidoreductase activity (GO:0016491) was enriched in all five GENIE3 runs and odorant binding (GO:0005549) in four of five runs performed on the same expression dataset. This recurrence supports computational stability of these enrichment signals rather than independent biological replication or demonstrated changes in physiological performance. Chemosensory performance, detoxification capacity, redox physiology, and survival were not directly measured; proposed consequences for these traits remain hypotheses. The redox signal aligns with the oxidative chemistry of melanization and cuticle tanning and with known links between mitochondrial function, thermal performance, and energy metabolism in bees. On the other hand, head-wide effects of a strong KO do not establish how naturally segregating, potentially tissue-specific *AmEbony* alleles act during abdominal cuticle pigmentation.

Together, our results establish *AmEbony* as a major-effect locus for abdominal pigmentation in East African honey bees. The close correspondence between *AmEbony* haplotypes and pigmentation, reinforced by the darker phenotype of CRISPR-edited bees, provides complementary population-genomic and experimental support for its central role. Head RNA-seq and GENIE3 further reveal reproducible transcriptional signatures involving mitochondrial metabolism, redox activity, and odorant-binding functions, expanding the functional landscape associated with *AmEbony* disruption. By integrating natural variation with experimental perturbation, this study provides a focused framework for identifying the functional variants and evolutionary forces shaping the striking pigmentation cline between East African highland and lowland bees.

## Supplementary Material

evag260_Supplementary_Data

## Data Availability

All newly sequenced honey bee genomes and transcriptomes were deposited in SRA with the BioProject accession number: PRJNA1445831.
