# Supplementary material for: Elevational Melanism in East African Honey Bees Involves a Major-Effect Locus
Source: Genome Biol Evol. 2026 Oct 14;18(10):evag260. doi: 10.1093/gbe/evag260 (PMC13720305; doi:10.1093/gbe/evag260)
Supplement: Supplementary file 1 [file evag260_supplementary_data.zip › Supplementary Information Legends.pdf]

**This PDF file includes:**

Legends for Supplementary Figure S1 to S9
Legends for Supplementary tables S1 to S7
Dataset link for SRA

**figures**

**Fig. S1.**

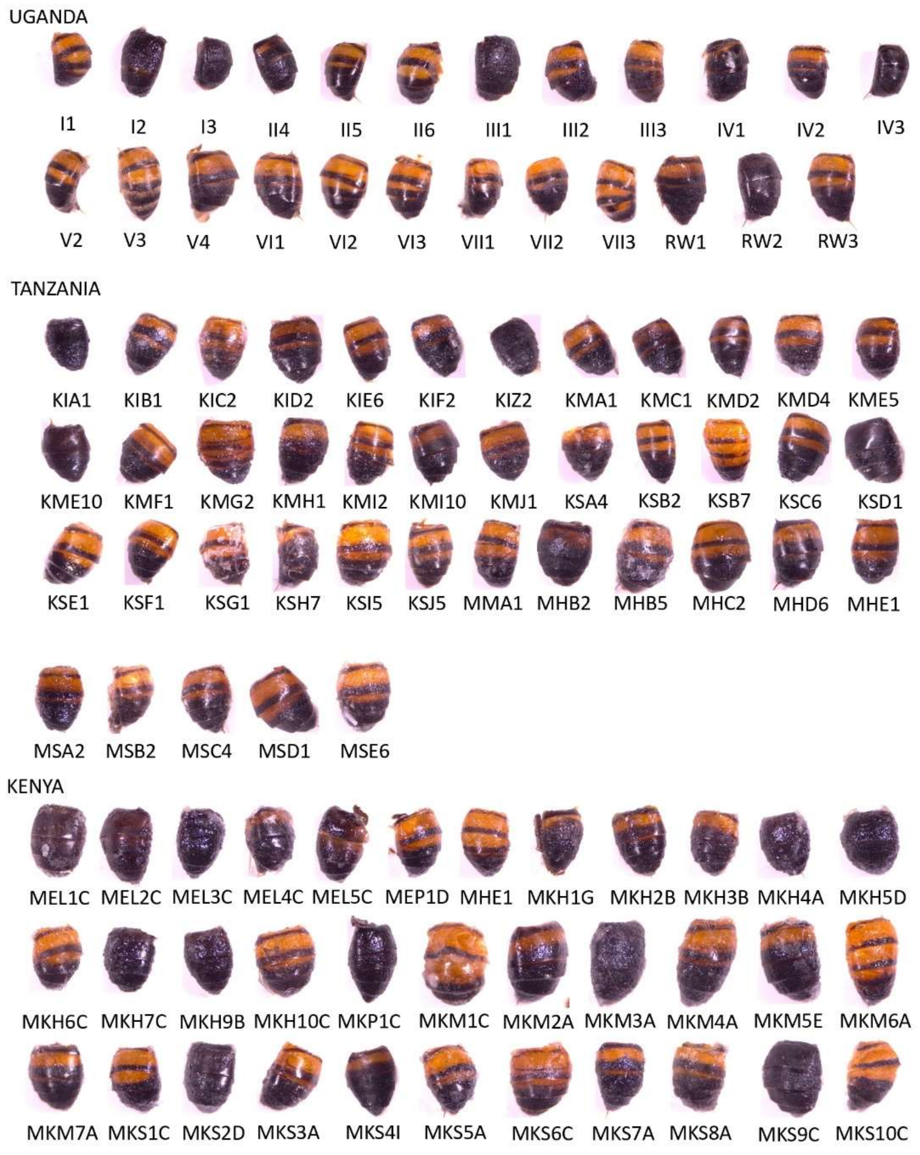


Photographs of the abdominal color patterns of all African honey bee specimens used in this study. The specimens shown here were sequenced and are organized by country and sample identifier.

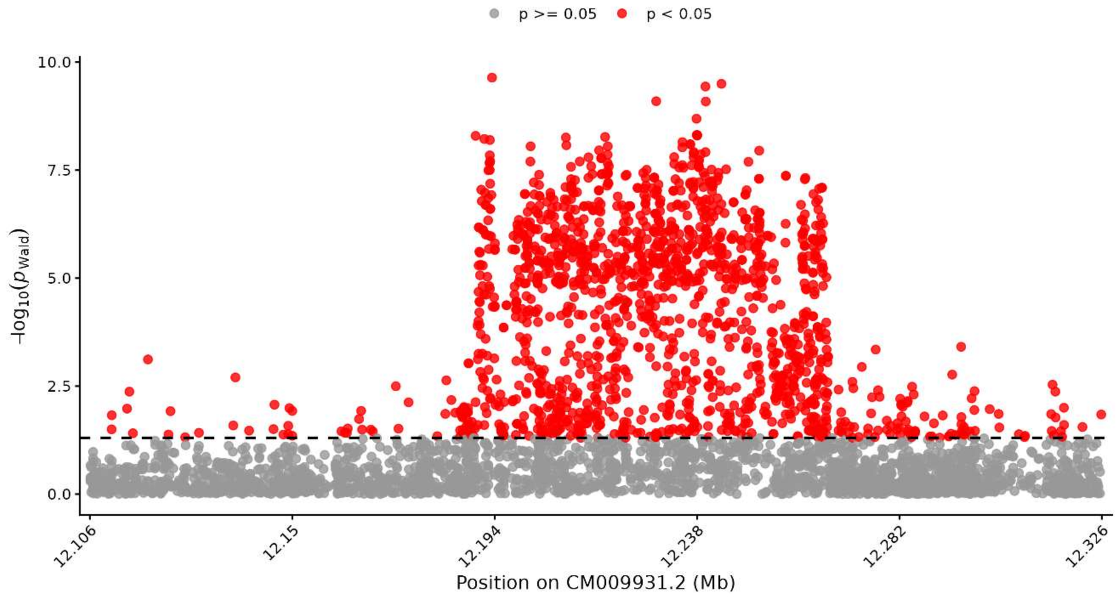


**Fig. S2.** Zoomed-in view of the GWAS association signal in the genomic region on chromosome 1 (CM009931.2) containing the main peak of significance. Each point represents a SNP plotted by genomic position and log10 adjusted p-value; red dots indicate SNPs above the significance threshold.

**Fig. S3.**

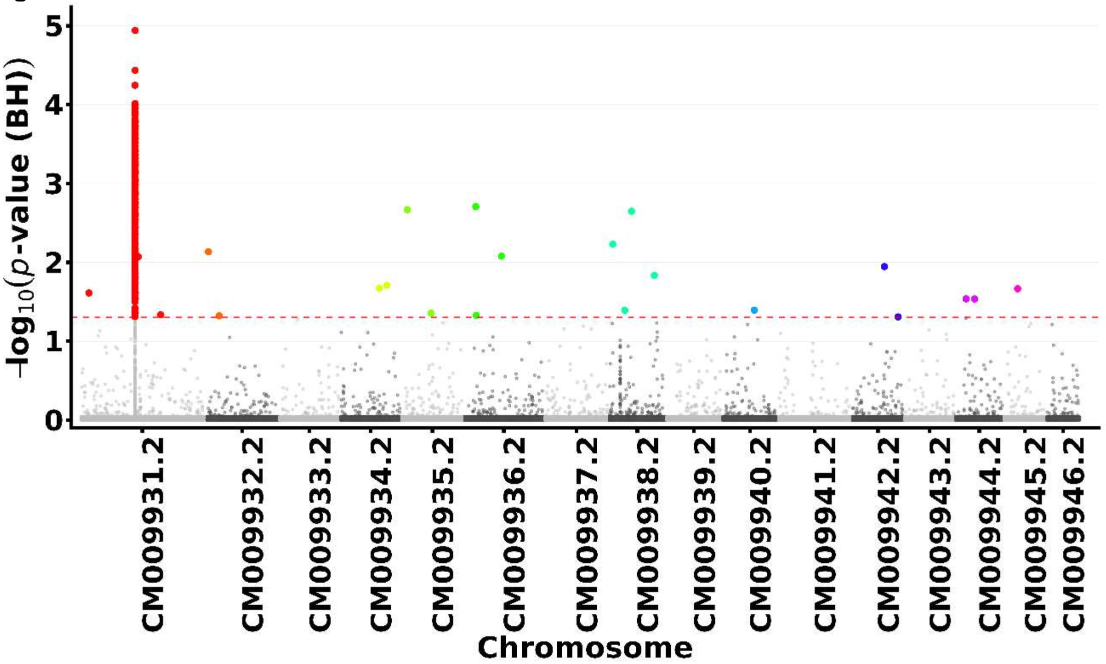


Results of the leave-one-chromosome-out (LOCO) analysis used as an independent validation of the GWAS signal. The strongest associations remain concentrated in the same genomic region, supporting the robustness of the detected peak.

Fig. S4.

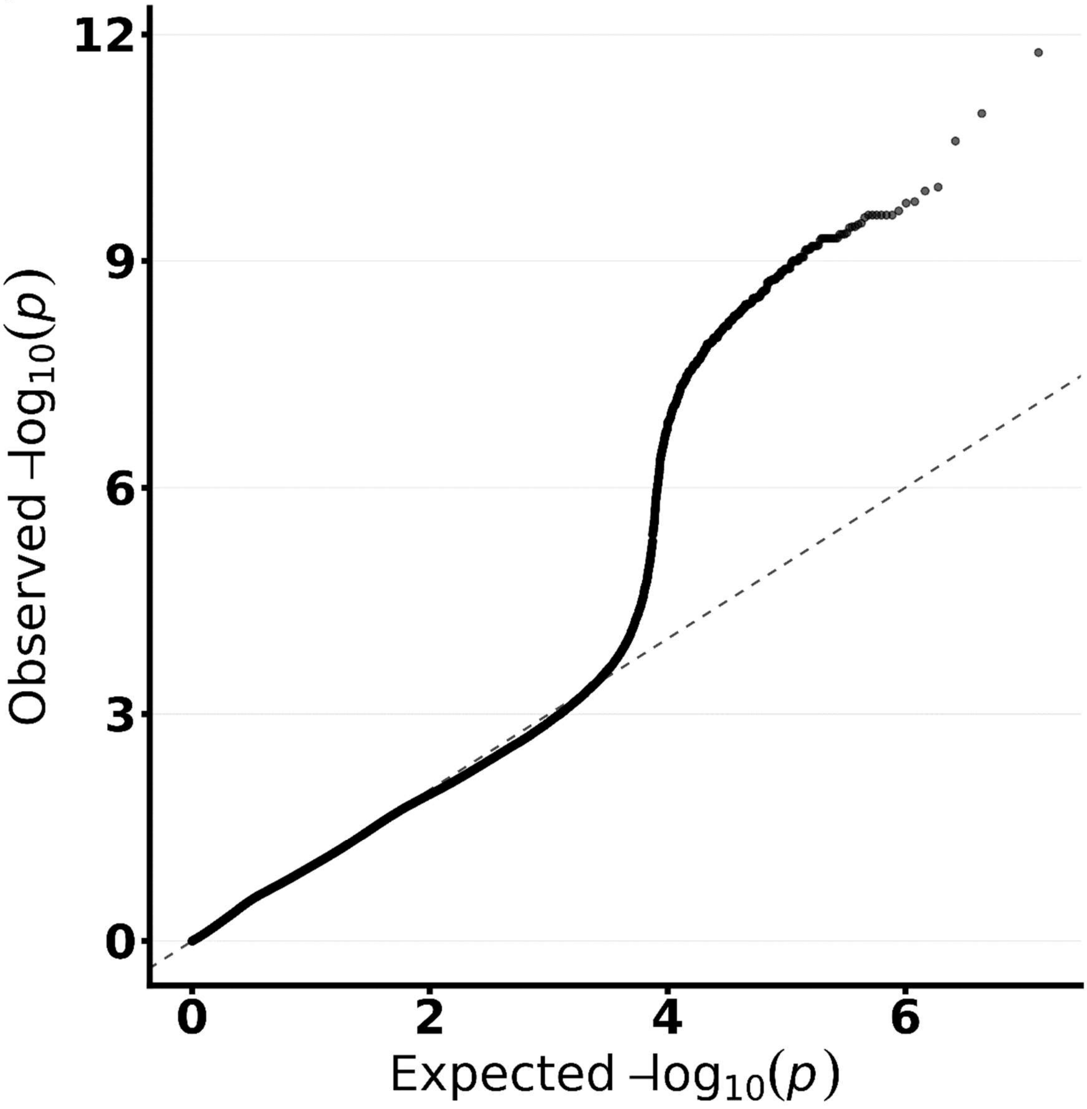


QQ plot for the LOCO analysis used to assess the distribution of association test statistics. The departure of the observed values from the expected null distribution is consistent with the presence of true association signals.

**Fig. S5.**

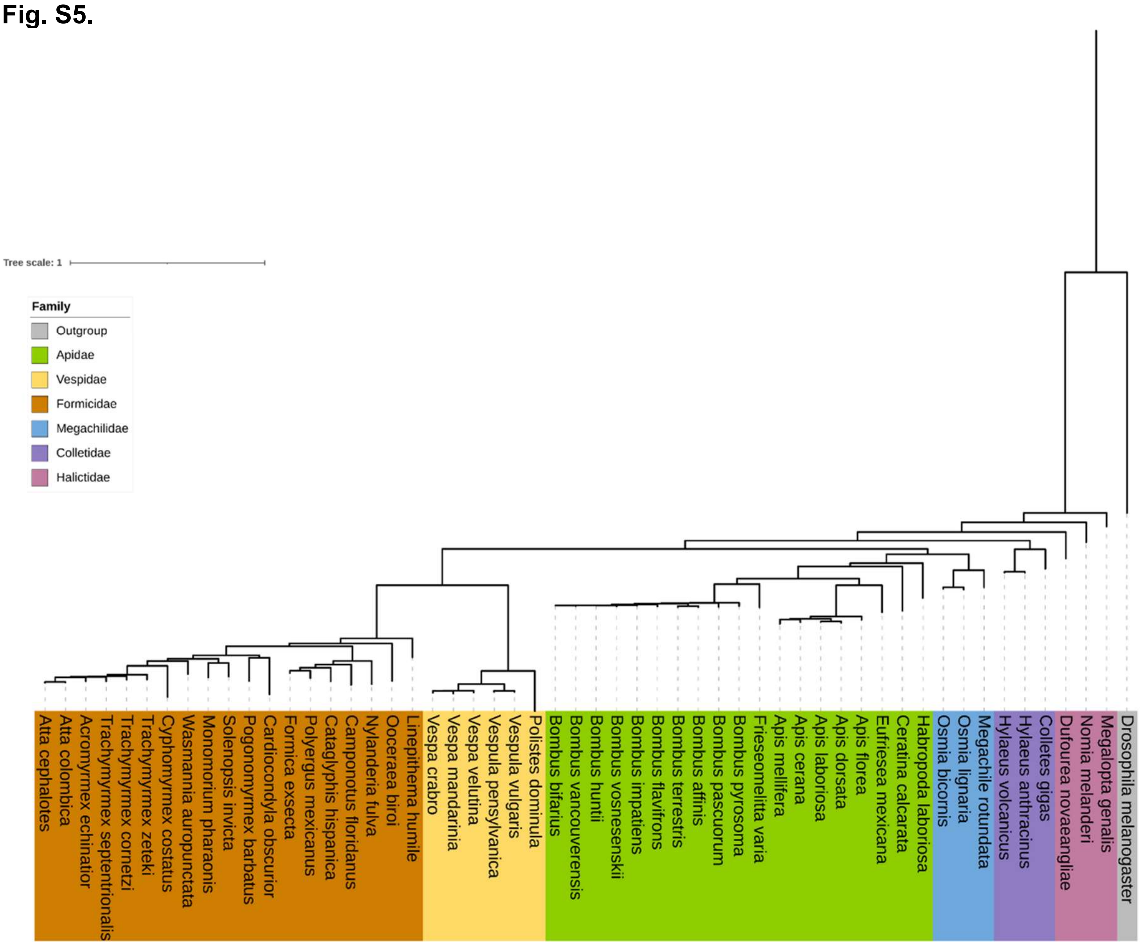


Gene tree of ebony orthologs across Hymenoptera, rooted with Drosophila melanogaster as the outgroup. Tip colors indicate clusters corresponding to different hymenopteran families.

**Fig. S6.**

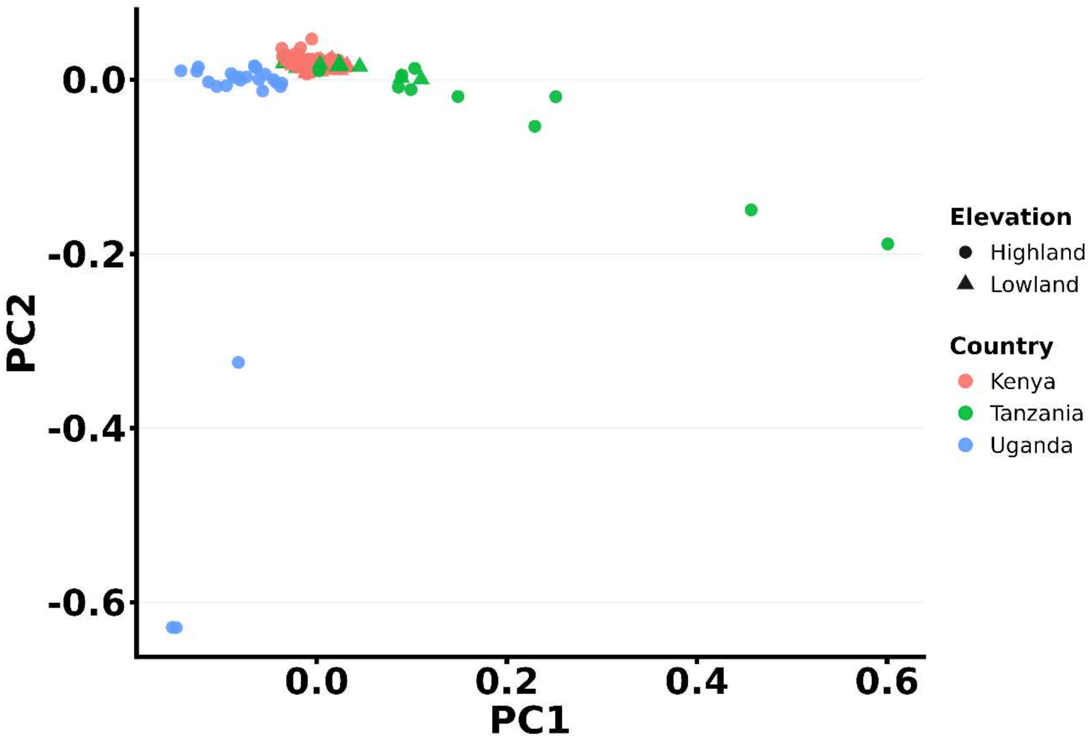


General Principal Component Analysis of the dataset used. By performing a PCA using Genome-Wide SNPs it is not possible to distinguish a specific pattern between population and pigmentation of the honey bees. Highland honey bees are represented by dots while lowland bees by triangles. Colors highlights populations included in this study.

**Fig. S7.**

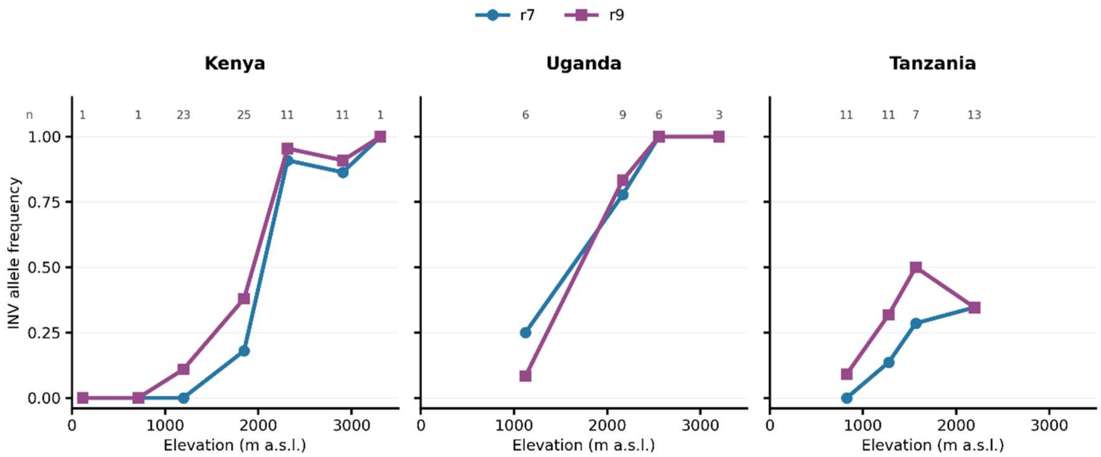


Elevational variation in r7 and r9 inversion allele frequencies in East African honey bees. PCA-inferred inversion frequencies are shown for Kenya, Uganda, and Tanzania in 500-m elevation intervals. Points indicate frequencies at each interval’s mean sampling elevation (r7, blue circles; r9, purple squares). Numbers indicate sample sizes.

**Fig. S8.**

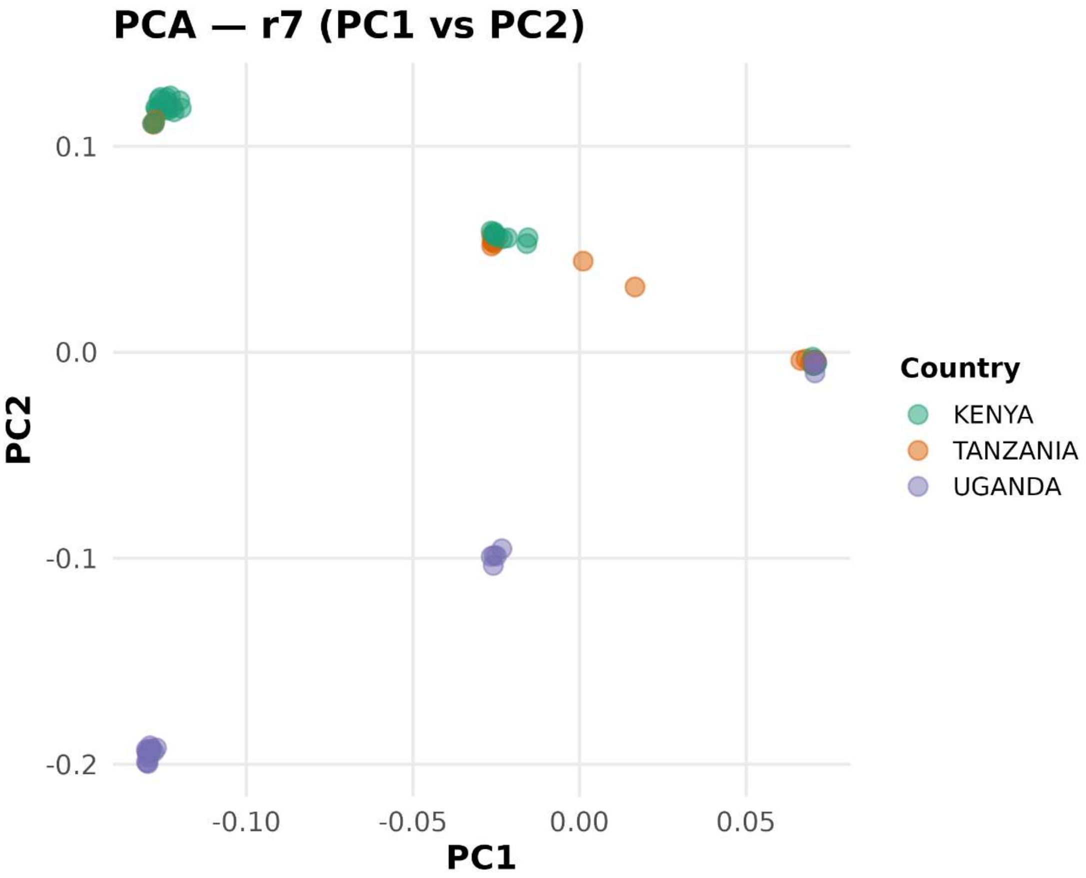


Principal component analysis of genetic variation across the r7 inversion region. Tanzanian samples cluster with inversion-associated groups, suggesting that this chromosomal inversion is also present in Tanzania.

**Fig. S9.**

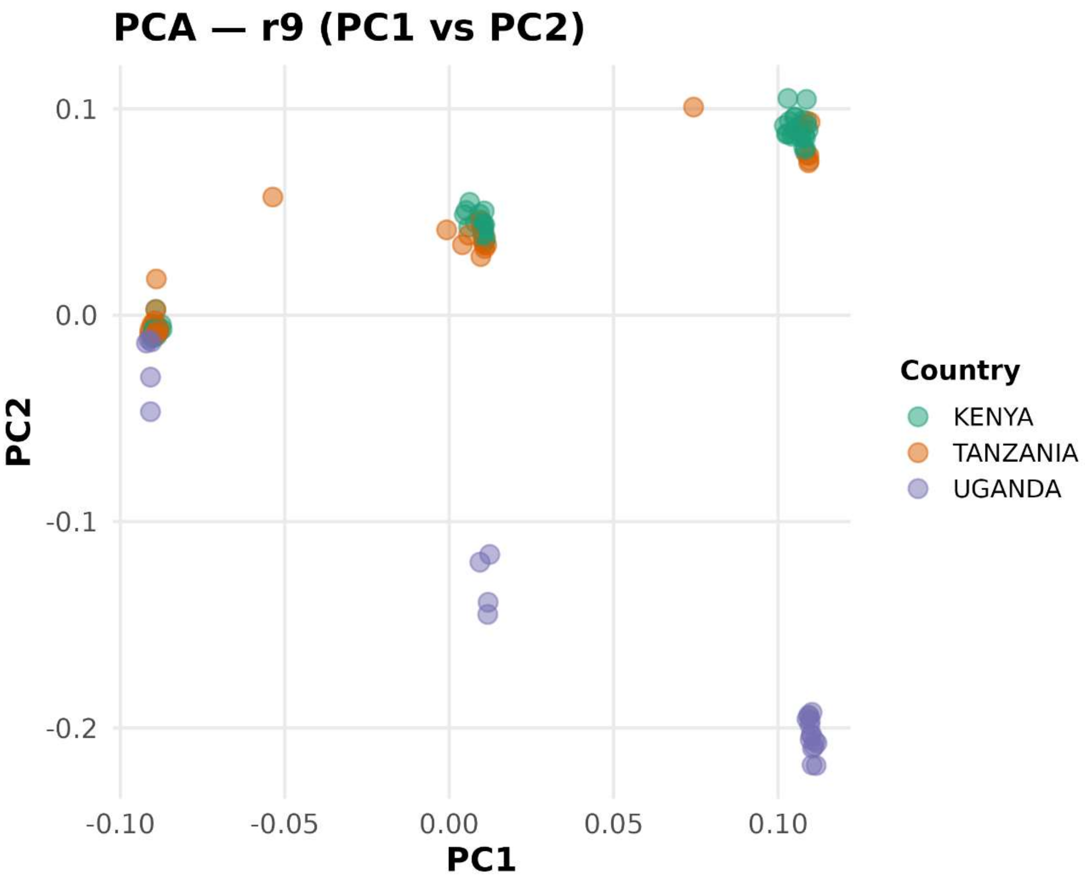


Principal component analysis of genetic variation across the r9 inversion region. Tanzanian samples cluster with inversion-associated groups, suggesting that this chromosomal inversion is also present in Tanzania.

**Fig. S10.**

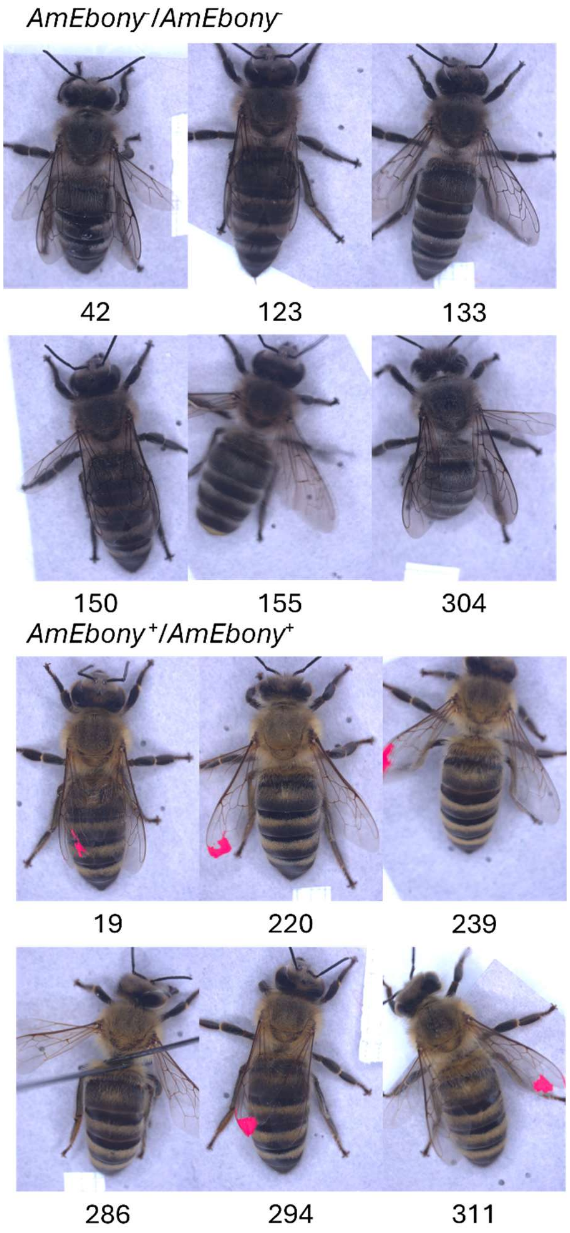


*Apis mellifera* individuals from the CRISPR/Cas9 experiment targeting *AmEbony* gene. The disruption of the gene resulted in markedly darker body pigmentation compared with wild type individuals

**Tables**

**Supplementary Table 1.** Coordinates of the sampled specimens and colonies from Kenya and Tanzania.

**Supplementary Table 2.** Pigmentation metadata of the dataset

**Supplementary Table 3.** Genes accession numbers and relative species and family information relative to the hymenopteran gene tree.

**Supplementary Table 4.** $F_{ST}$ and $D_{XY}$ comparison of significant SNPs on chromosome 1.

**Supplementary Table 5.** Annotation of Single nucleotide polymorphisms (SNPs) associated with the dark pigmentation and annotated with SNPeff.

**Supplementary Table 6.** Association between inversions r7 and r9 and the pigmentation phenotype. The association was computed using Chi-square test.

**Supplementary Table 7.** Results of significant modules and relative genes of the different GENIE3 runs.

**SRA Dataset link (for reviewers)**

**https://dataview.ncbi.nlm.nih.gov/object/PRJNA1445831?reviewer=474ivo0uj4pmk5t49i4f2d893m**
